# American Health Primers

**Published:** 1879-06

**Authors:** 


### AMERICAN HEALTH PRIMERS.

Dr. Keen, of Philadelphia, is editing a series of primers, which are designed to instruct readers as to the best methods of maintaining the health and vigor of both body and mind.

Without any doubt, the majority of our people sadly need instruction in preventive medicine. It is quite essential that this information be accurately and plainly given. The authors of the *American Health Primers* are the highest authorities in their specialties in America, and consequently the desired accuracy is insured. The following is a partial list of these little books, which are published by the well known house of Lindsay & Blakiston, Philadelphia: "I. Hearing, and How to Keep it"—by Charles A. Burnett, M. D., of Philadelphia, Surgeon in charge of the Philadelphia Dispensary for Diseases of the Ear, Aurist to the Presbyterian Hospital, etc.; "II. Long Life, and How to Reach It"—by J. G. Richardson, M. D., of Philadelphia, Professor of Hygiene in the University of Pennsylvania, etc.; "III. Sea Air and Sea Bathing"—by William S. Forbes, M. D., of Philadelphia, Surgeon to the Episcopal Hospital, etc.; "IV. The Summer and its Diseases"—by James C. Wilson, M. D., of Philadelphia, Lecturer on Physical Diagnosis in Jefferson Medical College, etc.; "V. Eyesight, and How to Care for It"—by George C. Harlan, M. D., of Philadelphia, Surgeon to the Wills (Eye) Hospital; "VI. The Throat and the Voice"—by J. Solis Cohen, M. D., of Philadelphia, Lecturer on Diseases of the Throat in Jefferson Medical College; "VII. The Winter and its Dangers"—by Hamilton Osgood, M. D., of Boston, Assistant Editor *Boston Medical and Surgical Journal;* "VIII. The Mouth and the Teeth"—by J. W. White, M. D., D. D. S., of Philadelphia, Editor of the *Dental Cosmos*, "IX. Our Homes"—by Henry Hartshorne, M. D., of Philadelphia, formerly Professor of Hygiene, in the University of Pennsylvania; "X. The Skin in Health and Disease"—by L. D. Bulkley, M. D., of New York, Physician to the Skin Department of the Demilt Dispensary and of the New York Hospital; "XI. Brain Work and Overwork"—by H. C. Wood, Jr., M. D., of Philadelphia, Clinical Professor of Nervous Diseases, University of Pennsylvania, etc. Other volumes are in preparation, including the following subjects: "Preventable Diseases," "Accidents and Emergencies," "Towns We Live In," "Diet in Health and Disease," "The

Art of Nursing," "School and Industrial Hygiene," "Mental Hygiene," etc. They will be 16mo in size, neatly printed on tinted paper, and bound in paper covers. Price, 30 cents, flexible cloth, 50 cents.

